# Yellow Fever Commission

**Published:** 1878-12

**Authors:** 


### YELLOW FEVER COMMISSION.

In the November number of this JOURNAL we alluded, in a short article, to the forthcoming report of this Commission. Our readers are already familiar with the manner of its organization. It is known that Dr. Woodworth, Surgeon-General of Marine Hospital Service, appointed Dr. Bemis, of New Orieans, Dr. Cochran, of Mobile, and Dr. Howard, of Baltimore, to form a commission for the purpose of investigating the origin and spread of yellow fever. Col. Hardee was also appointed sanitary engineer of the Commission.

Dr. S. M. Bemis, chairman, was joined in New Orleans by the other two members early in October, and the work of investigation commenced.

The great object seems to have been to prove that yellow fever is exotic — that the disease was imported to New Orleans, and transported thence to other places on the Mississippi river. While we do not know the previous views of all those concerned in the investigation, it is certain that Dr. Woodworth, creator of the Commission, had expressed the opinion that yellow fever is exotic, is always imported to this country, is not indigenous, and has no local origin here.

The work was commenced by a survey of the fever portions of the city, and the location of the first cases noted on the map. The history given by the residents of New Orleans, upon which the Commission base their conclusions, and upon which they rely for proof of importation, is found in the following extract from their report, made through Dr. Bemis, the chairman, to the National Health Association at its meeting in Richmond, Virginia:

"On the 23d of May, the Emily Souder arrived at New Orleans with her purser sick at the time of her arrival. This man, whose name was Clark, was carried to Claiborn street, near the corner of Blenville, at a point conspicuously designated on the map of New Orleans. At this house he died on the 25th of May. The death was returned by the attending physician as one from malarial fever. For testimony establishing the fact that this was a case of yellow fever, I refer the association to Dr. Cochran's notes. Another of the crew of the Souder by the name of Elliott took sick May 24th, at the corner of Girard street and from which place he was taken to the Hotel Dieu, May 27th, and died on the 30th. The Commission deemed it important, as a first step in their work, to ascertain whether such connection existed between these imported cases and those occurring in New Orleans as to authorize them to declare that they afforded the fact of infection from which the disease afterwards spread in the city. We were compelled to leave New Orleans before this point in our investigation had been satisfactorily accomplished. Enough was developed, however, to render it probable that a connection, as yet untraceable, does exist between the cases, Clark and Elliott, and the first cases among the citizens of New Orleans. It is proper to add, at this point, that the Commission received a number of letters and some verbal statements purporting to give information respecting violations of quarantine by fruit vessels and other ships entering the port of New Orleans from infected ports. Every effort which could be made in the limited time we had for work in New Orleans was put on foot to ferret out the facts connected with such alleged infringement of quarantine laws. We obtained a sufficient amount of testimony to justify a belief that one or more cases of yellow fever had occurred in the city, probably in the month of June, under circumstances which rendered it altogether poosible that they had been brought to the city by conveyances as yet unknown (see testimony of Drs. Cochran and Bemis) from the time that the disease made its appearance in this form of series or groups of cases, each having connection with some other, either by personal association or from exposure in the same locality. We have located ten cases on a map, which comprise all the reported cases which occurred in July. To this map have been added a group of cases occurring during the first ten days of August, because of their importance when studied in connection with the epidemic which subsequently occurred in Canton, Mississippi."

Now, in this first, grand, decisive step of the investigation, we assert that the effort to sustain the importation theory has proved an utter and virtually acknowledged failure! The case of fever landed at New Orleans on the 23d of May belonged to the crew of a vessel hailing from some port not known or not necessary to be mentioned. A little information as to whether the crew had been exposed to the causes of disease at Havana, New York or Baltimore might have given more general satisfaction. The attending physician reported the case to be one of malarial fever, in which is generally included remittent, simple intermittent or malignant intermittent, called congestive fever. The Commission ascertained that the physician in attendance was mistaken in his diagnosis, but from whom and in what manner we are not informed. Suppose it be true that the doctor was mistaken, and that two *bona fide* cases of yellow fever were thrown into the populous city of New Orleans, and suppose, for the sake of the argument, that they were contagious, is it not passing strange that from this hot-bed of disease, only "one or more cases" should occur during the whole of the following month of June, and "it altogether possible that *they* had been brought to the city by conveyances as yet unknown?" Indeed, is it not unreasonable to suppose that after thirty days the disease should spread from the two seamen? To say the least of it, this is certainly an immense period of incubation!

The Commission certainly confess their failure to establish even a "connection untraceable," or "probable," when they say, alluding to the connection between these and subsequent cases: "We were compelled to leave New Orleans before this point in our investigation had been satisfactorily established."

Upon "this first step" the whole question depends, and we view it as a serious misfortune to the country that the Commission were "compelled to leave" without coming to, and expressing, a definite conclusion upon undeniable facts presented. The report goes out to the world as settling the question of importation by selected talented and scientific physicians, when really no positive conclusion is had. Better that no report had been made than one of possibilities and probabilities, with a decided leaning to what was and could not be proved.

The importation theory is adopted by government officials, and the Commission endeavor, though unsuccessfully, to prove it true. With these authorities before him, the President of the United States feels called upon to suggest, in his recent message to Congress, the importance of their prompt attention to the subject of quarantine. To show that he is thoroughly impressed with the popular idea of transportation of the disease which is so zealously sought to be made general, the President says of yellow fever: "It was rapidly spread by fugitives from the infected cities and towns." * * Now, without sufficient proof of such "spread," the legislative department of the government is to be called on for the appropriation of large sums of money to consummate the project of certain officials for quarantine, the effects of which will work detriment to commerce and personal comfort, without compensating benefit to any.

The zealous, self-sacrificing gentlemen of the Commission labored, doubtless, for the benefit of mankind, but evidently have overlooked an important channel of investigation. Traveling, infectious or contagious yellow fever has engaged their thoughts, to the exclusion of the deadly poison originating in the swamps, marshes and pools of the Mississippi and its tributaries the past summer. An unusual overflow, and intensely hot weather, has led to the production of malignant malarial poison along the course of this river which receives the washings of so large an area of fertile cultivated land.

The Commission, it seems, entirely neglected to investigate the surroundings of infected districts. Were there not indigenous cases of undoubted malarial fever, preceding and during the entire epidemic of yellow fever in New Orleans? This fact should certainly have been mentioned to show the usual co-existence of and connection between yellow fever and other forms of malarial fever, such as intermittent, remittent and congestive.

Had there been appointed a member on this Commission without preconceived settled views against the local origin of yellow fever, these facts would, in all probability, have been mentioned in the report.

The Commission have certainly neglected to report on the probable local cause of disease in New Orleans and other points, except as to the want of sanitary attention to streets, at some points. Why, we ask, were not the local causes for the unusual amount of malarial poison at all the points where yellow fever occurred, mentioned by the Commission? Common sense, humanity and the general interests of this country demand such investigation, and such report. This and other epidemics of yellow fever, we assert, follow and are traceable to unusual cause of malaria where they occur, and that milder forms of malarial fever, such as simple intermittent, remittent, as well as malignant intermittent, accompany yellow fever. Savannah, in 1876, had the surroundings suited to the production of malaria, and the evidences of its existence, in unusual amount, without a single fact proving importation of yellow fever, as the Board of Health were forced to admit. Brunswick, the same year, had the same conditions, to-wit: overflow from excessive rains, and excessively hot weather; and every variety of malarial disease, including yellow fever was the result. Importation was there said also to be proved, by various circumstances; one of which was the boarding of a ship by a seamstress, who contracted yellow fever the next day. This or any other proof is sufficient for those whose minds are already made up on the subject, and are determined to believe nothing but importation.

If the assiduous labors devoted to the discovery of connection between foreign and indigenous cases had been divided so as to give some attention to the other side of this question, the cause of humanity would have been more profitably served. The almost yearly transportation, and even importation, of yellow fever cases, to locations where no malarial cause of disease exists, without communicating it to others, is a fact that should be prominently noted by a commission, whose report is to govern the sanitation of this country in regard to a disease so destructive to life as yellow fever. Hundreds of non-malarious places have been made the test of personal infection by having yellow fever cases brought to them, and the disease *did not* "*spread.*" In a fair and earnest search for truth, such facts should be taken into consideration. For twenty-five years, Atlanta has been made the refuge of yellow fever sufferers, when epidemics occur on the coast and large rivers of the South. Many of the refugees have had the fever here. Some have died with black vomit, while others have recovered; but in no instance has the disease been communicated, however closely associated citizens of this place may have been with the sick. Much evidence of this kind can be secured without any trouble to the seeker after truth on this subject.

If, then, the disease be not communicated in a non-malarious locality, why should its spreading be attributed to personal infection or contagion where malarial cause exists? Travel and various kinds of intercommunication are carried on between various infected and healthy points at all times, and the fever prevails every year, but is not carried and does not exist in Savannah, Augusta and various places on the Mississippi river, unless the condition of these localities favor the production of the poison. Then, if the cause must be there, in order to the development, why conclude that the disease is imported or carried from some other place? No "spark" is necessary to "ignite the tinder." When malaria is in sufficient quantity and of sufficient virulence, malignant fever in the form of congestive, yellow, or hæmaturial, will be found.

In conclusion, we have this to say: In order to avoid danger, it is necessary, first, to know where it lies, and next, the mode of escape. Yellow fever is evidently contracted by personal infection or contagion, or by exposure to malarial poison. If the former be the correct theory, the danger may be avoided by preventing contact with subjects of the disease; *if the latter, the conditions necessary to the production of the malaria must be changed, or the inhabi-*

*tants must flee from the poisoned locality.* In the settlement of this question, there is something more involved than the solution of an abstract scientific problem. Thousands of valuable lives hang upon the decision arrived at. If to quarantine we must look for safety, it must be made perfect; and, on the other hand, if local origin is settled upon as true, and the generation of the poison cannot be prevented, safety depends upon flight. Error in conclusion, therefore, works mischief. If quarantine is relied on, no sanitary measures will be adopted, and the subtle poison slowly but surely does its work of death.

In Congress, already, has a committee of the Senate been appointed to join a like one of the House "to inquire into the cause of yellow fever, what legislation is necessary to prevent its introduction into the country, etc." We earnestly implore this committee to require and examine facts on the other side of this question before deciding upon measures directed solely against importation and transportation. Close observers have proven the local origin of this fever, and such proof should not be ignored in reports of medical men, upon which measures of relief are to be instituted by the government. The useless expenditure of money on quarantine will be a small matter compared with the suffering and death which will result from the error of personal infection being acted on, leaving the people an easy prey to the enemy at home, by having their attention called away from the true origin of the disease.

---

We have just received a package containing preparations of pepsin pancreatine and malt. The revenue stamps on the bottles indicate some special privilege conferred by the government which we do not very well understand. It cannot be that a patent for the exclusive use of these useful articles has been secured, for all pharmacists make them, and all apothecaries dispense them. It is presumable that the exclusive right has been granted, as a pharmacist, to unite these articles in certain proportions for the trade under a definite name.

Now, of this manner of dealing among druggists with each other we have nothing to say, if the public and physicians are not to be affected unfavorably by it. The names and amounts of the articles associated in each bottle are given, but though patented in this form, the right of a physician to order from an apothecary a prescription of these remedies, in the exact proportion given on the bottle, is certainly not affected. The preparations are well tasted, and the proportions, as given on the bottles, well suited for use.

The Medical Code of Ethics condemns patent medicines, and, we think, properly. Every thing known by a physician is free to all physicians and to the public. Secret remedies and the exclusive right to use any particular medicine work detriment to the public, and are therefore reprehensible.

